# Tapering Antidepressants While Receiving Digital Preventive Cognitive Therapy During Pregnancy: An Experience Sampling Methodology Trial

**DOI:** 10.3389/fpsyt.2020.574357

**Published:** 2020-10-22

**Authors:** Marlies E. Brouwer, Nina M. Molenaar, Huibert Burger, Alishia D. Williams, Casper J. Albers, Mijke P. Lambregtse-van den Berg, Claudi L. H. Bockting

**Affiliations:** ^1^Department of Clinical Psychology, Utrecht University, Utrecht, Netherlands; ^2^Department of Psychiatry, Amsterdam University Medical Centres, Location AMC, University of Amsterdam, Amsterdam, Netherlands; ^3^Departments of Psychiatry and Child and Adolescent Psychiatry/Psychology, Erasmus University Medical Centre Rotterdam, Rotterdam, Netherlands; ^4^Department of Psychiatry, Icahn School of Medicine at Mount Sinai, New York, NY, United States; ^5^Department of General Practice, University Medical Centre Groningen, University of Groningen, Groningen, Netherlands; ^6^Faculty of Science, School of Psychology, The University of New South Wales, Sydney, NSW, Australia; ^7^Heymans Institute for Psychological Research, University of Groningen, Groningen, Netherlands; ^8^Institute for Advanced Study, Netherlands Institute for Advanced Study in the Humanities and Social Sciences, Royal Netherlands Academy of Arts and Sciences, University of Amsterdam, Amsterdam, Netherlands; ^9^Centre for Urban Mental Health, University of Amsterdam, Amsterdam, Netherlands

**Keywords:** pregnancy, preventive cognitive therapy, antidepressants, tapering and discontinuation, offspring, experience sampling methodology (ESM), proof-of-principle

## Abstract

**Background:** Previous studies indicated that affect fluctuations, the use of antidepressant medication (ADM), as well as depression during pregnancy might have adverse effects on offspring outcomes. The aim of the current proof-of-principle study is to explore the effect of tapering ADM while receiving online preventive cognitive therapy (PCT) on pregnant women and the offspring as compared to pregnant women continuing ADM.

**Objectives:** We sought to compare positive and negative affect fluctuations in pregnant women receiving online PCT while tapering ADM vs. pregnant women continuing ADM, and to investigate if affect fluctuations in early pregnancy were related to offspring birth weight.

**Method:** An experience sampling methodology (ESM)-trial ran alongside a Dutch randomized controlled trial (RCT) and prospective observational cohort of women using ADM at the start of pregnancy. In the ESM-trial fluctuations of positive and negative affect were assessed in the first 8 weeks after inclusion. Recurrences of depression were assessed up to 12 weeks post-partum, and birth records were used to assess offspring birth weight. The RCT has been registered at the Netherlands Trial Register (NTR4694, https://www.trialregister.nl/trial/4551).

**Results:** In total, 19 pregnant women using ADM at start of their pregnancy participated in the ESM-trial. There were no significant differences in positive and negative affect fluctuations, nor recurrence rates between women receiving PCT while tapering ADM vs. women continuing ADM. We found no association between affect fluctuations, pre-natal depressive symptoms, and birth weight (all *p* > 0.05).

**Conclusion:** This explorative study showed that tapering ADM while receiving online PCT may protect pregnant women against recurrences of depression and affect fluctuations, without affecting birth weight. There is a high need for more controlled studies focusing on tapering ADM with (online) psychological interventions during pregnancy.

## Introduction

Major depressive disorder (MDD) is a highly disabling and recurrent disorder that affects people worldwide, including pregnant women. Women with a history of mental disorders are at increased risk of perinatal depression (1, 2). Reported recurrence rates of MDD during pregnancy range widely from 2.5 to 68%, depending on the population, treatment, and follow-up period (3–7). MDD and recurrences in the perinatal period can place women and their offspring at risk of short- and long-term psychological and somatic problems, including low birth weight, pre-term birth, and shorter gestational age, which in turn are associated with psychopathology at later ages (8–10), development of MDD, anxiety, and developmental disorders (11–15). Birthweight and gestational age can hence be perceived as first indications of infant's future (mental) health. It is presumed that psychological and pharmacological treatment of pre-natal common mental disorders can mitigate associated adverse effects in offspring, yet strong evidence for the prophylactic benefits of (preventive) treatment is limited (16, 17).

Current treatments to prevent recurrences of MDD during pregnancy include the use of antidepressants medication (ADM). ADM use during pregnancy is estimated to be around 2–8% for various psychiatric disorders (18–21), however, reviews have linked pre-natal ADM use to negative offspring outcomes, including lower birth weight, shorter gestation, development of psychopathology, and cardiovascular problems (22–24). Alternatively, sequentially offering a specific psychological relapse prevention treatment after (partial) remission (i.e., mindfulness-based cognitive therapy [MBCT], preventive cognitive therapy [PCT], or well-being therapy [WBT]) protects against recurrences of MDD, when compared to active (e.g., ADM or treatment as usual [TAU]) or non-active (waitlist) control groups (25–35). Psychological prevention treatments have furthermore been demonstrated to be effective in lowering depressive symptoms during pregnancy (17, 36), and may lower the risk of recurrence of MDD (37). In a recent randomized controlled trial (RCT) (38–41), we investigated the efficacy of PCT with gradual, guided discontinuation of ADM compared to ADM continuation among formerly depressed women taking ADM at the start of their pregnancy. The women (*n* = 44) were followed throughout pregnancy and up to 3 months post-partum to evaluate recurrences of MDD and the potential impact of the interventions on mother and offspring. There was no evidence that PCT with gradual discontinuation of ADM during pregnancy altered the risk of recurrence of MDD as compared to continuation of ADM (41). This study provides first evidence that preventive therapy while tapering ADM may be a viable alternative to ADM usage for both the formerly depressed pregnant women and their offspring.

Early identification of women at risk and subsequent prevention of relapse or recurrence of MDD in the perinatal period are hence of importance. Previous research showed that certain patterns of variability of affect within individuals may indicate that the individual is prone to depressive relapse or recurrence (42–44). Some studies showed that low positive affect and high negative affect were related to increased depressive symptoms, and increased risk of recurrence of MDD (45–47). On the other hand, a case study and a study in depressed patients (*n* = 93) showed that the inertia of affect, i.e., the lack of variability, were indicators that a person relapsed into depression (43, 48), although this was not confirmed in a recurrently depressed patient sample (*n* = 42) (49). The variability of affect is nevertheless believed to be clinically relevant and warrants further investigation.

Affect and affect fluctuations are indeed important to consider in the context of pregnant women and their offspring since previous studies showed that increased positive affect may prevent pre-term birth, although without changing birth weight (50), and that positive and negative affect fluctuations are associated with poorer offspring outcomes such as disturbed fetal physiology [e.g., decreased fetal heart rate and intrauterine artery flow (51)]. At the same time, symptoms of depression and anxiety, and stress levels commonly vary throughout pregnancy (52–54), which in turn are associated with negative affect (reported by the mother) in 1–7 year old children (55), and delayed child development (54). These studies highlight that fluctuations in positive and negative affect and offspring outcomes need to be monitored, especially during relapse prevention interventions.

Experience Sampling Methodology (ESM) (56), which has been successfully used in previous studies (44, 47, 49), provides the opportunity to monitor affect fluctuations and the impact on offspring by multiple semi-random assessments through a mobile telephone application during the day. ESM-trials allow researchers to profoundly investigate day-to-day affect and mood (changes) and early warning signs of depressive relapse in pregnant women. ESM surpasses the problem of traditional self-report questionnaires, where there is an increased risk of a recall bias. For example, self-report questionnaires such as the Edinburgh post-natal depression scale, include questions regarding mood across 1 full week, while it is known that mood symptoms fluctuate throughout the week, or even 1 day (57). In addition, the use of ESM permits researchers to answer research questions by investigating less participants while gathering more information than in traditional designs, which enables including difficult-to-reach populations in research, like the population in the previously described RCT (41).

The question remains whether pregnant women receiving PCT while tapering ADM have more affect fluctuations as compared to pregnant women continuing ADM. Furthermore, it is currently unknown whether affect fluctuations can predict the return of depressive symptoms or recurrence of MDD in pregnant women, and predict offspring health (as measured in terms of birth weight, with and without correction for gestational age). Offspring birth weight is important to investigate due to its relation with future developmental, mental, and somatic disorders. Using an ESM-design alongside the previously described RCT and observational cohort (38–41), the aims of the current study are to (1) explore whether there are more fluctuations in negative and positive affect in pregnant women receiving PCT while tapering ADM vs. continuing ADM; (2) explore whether affect fluctuations predict subsequent recurrences and/or pre-natal depressive symptoms; and (3) explore whether affect fluctuations predict lower offspring birth weight.

## Materials and Methods

### Design

This article focuses on the ESM-trial run alongside an RCT and an observational cohort. Participants for the ESM-trial were drawn from an RCT [“Stop or Go study” (39)] and a prospective, longitudinal, observational cohort (38, 40), to investigate the effects of PCT while tapering ADM vs. the continuation of ADM. All studies were approved by the Medical Ethical Committee of the Erasmus Medical Center Rotterdam, the Netherlands (MEC-2014-505). All participants provided written informed consent prior to participation.

The RCT protocol has been pre-registered (Netherlands Trial Register: NTR4694) and published (39). The observational cohort has been described in other publications (38, 40) and the protocol has not been pre-registered, as this group of participants was a reference group for the RCT. The ESM-trial started at a later stage of the studies due to the development and coordination of the mobile application, and is therefore not reported in the protocol article.

### Participants

#### ESM-Trial

Participants from the RCT and observational cohort were invited to participate in the ESM-trial after providing written informed consent for the RCT or observational cohort. Participant in-and exclusion criteria and procedures for the RCT and observational cohort are described below. There were no additional criteria to participate in the ESM-trial.

#### RCT and Observational Cohort

To participate in the RCT or cohort, women needed to (1) be <16 weeks pregnant; (2) use an ADM at the start of the pregnancy, such as selective serotonin reuptake inhibitors (SSRI), or selective noradrenaline reuptake inhibitor (SNRI); and (3) be proficient in Dutch and/or English. Additionally, to participate in the RCT, pregnant women needed to (1) have a history of at least one MDD episode according to the DSM-IV Axis-I (58); (2) be in remission or recovery, i.e., not have a diagnosis of MDD according to the DSM-IV Axis-I criteria since at least 4 months before participation; (3) be willing to be randomized to either PCT with tapering of AMD (“Stop”) or continuation of ADM (“Go”); and (4) have a singleton pregnancy.

### Procedures

#### ESM-Trial

All participants in the RCT and observational cohort were invited to participate in the ESM-trial. Inclusion for the ESM-trial was from August 2016 to February 2018. The ESM-trial started immediately after the baseline assessments and (when applicable) randomization. As a result, the assessments were conducted at the same time as when the participants received PCT and tapered ADM, or continued ADM.

The ESM-trial consisted of questions that were sent in the first 8 weeks of study participation through the mobile telephone with the use of a pre-installed study application using the Tempest platform (59). This application was programmed on the participants' own phone, or on a study-smartphone that was available during study participation. It was programmed to send pre-defined questions 5 days a week, 5 times a day, for 8 consecutive weeks. The triggers were set semi-randomly between 9:00 a.m. and 7:00 p.m., triggering once every 100 min, including a pause of 10 min minimum between triggers. Time to complete the questions was 1–2 min. Participants were instructed to respond to the trigger as soon as possible. To monitor reasons for non-response, a research assistant contacted the participants to discuss problems or questions regarding the application.

#### RCT and Observational Cohort

**Figure 1:**
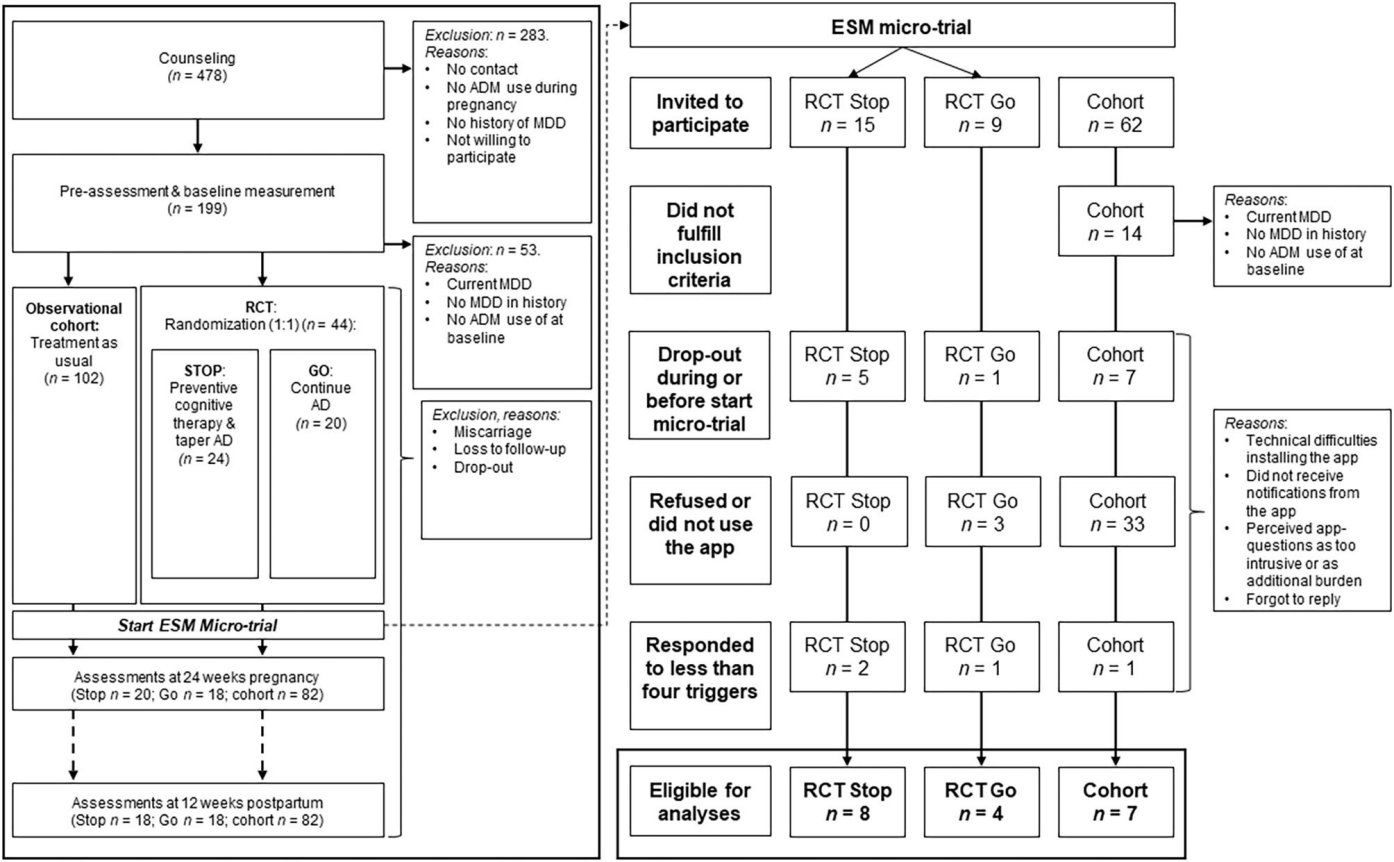
Flow chart of study participation and exclusion (reasons). RCT, Randomized controlled trial; RCT Stop, RCT group receiving preventive cognitive therapy while tapering ADM; RCT Go, RCT group continuing ADM; ADM, antidepressant medication; ESM, experience sampling methodology.

Participants were recruited between April 2015 and February 2018 during their first pre-natal visits at the midwifery practices or hospitals in the Netherlands, through general practitioners, psychiatrists, or through advertisements in (social) media. After study researchers received contact information of potential eligible pregnant women, the study researchers counseled the women about the RCT and the observational cohort. After the counseling and a waiting period to think about participation, pregnant women decided to either (1) not participate; (2) participate in the RCT; or, (3) participate in the observational cohort. After this decision and written informed consent, there was a baseline assessment by means of a structured clinical interview through telephone [SCID-I DSM-IV (58)], and a self-report questionnaire. If participants met inclusion criteria and were willing to participate in the RCT, they were randomized into one of the two groups: PCT with tapering of ADM (“Stop”) or continuation of ADM (“Go”). Alternatively, the pregnant woman started participating in the observational cohort, where women decided themselves which relapse prevention strategy they wanted to use. Hereafter, women were assessed with questionnaires and interviewed at 24 and 36 weeks of pregnancy, and 4 and 12 weeks after the due-date. See Figure 1 for a flowchart of all studies.

### Interventions

#### PCT and Tapering ADM

Women assigned to the “Stop” -group received online PCT and tapered ADM. The PCT was provided by trained psychologists and consisted of 8 weekly individual sessions using an online telehealth app. In short, PCT uses techniques focused on dysfunctional beliefs and schema using cognitive challenging techniques, including phantasy (activation of positive network), enhance the recall of specific memories of positive experiences, positive feelings and thoughts, and formulating relapse prevention strategies (60). To taper the ADM, the woman was referred to a trained psychiatrist or general practitioner who then guided the tapering. Tapering schedule was based upon an expert-based discontinuation protocol, individual preferences, and drug characteristics. Participants were not allowed to receive another form of psychotherapy on a regular basis during PCT, i.e., first 8 weeks (during the ESM-trial).

#### Continuation of ADM

Pregnant women who were assigned to the “Go” -group continued the use of ADM as instructed by their prescribing doctor (care as usual).

#### Observational Cohort

Women in the observational cohort decided themselves which prevention strategy they used (care as usual). This therefore includes women who tapered ADM without PCT.

### Measurements

A full overview of this schedule and all assessments can be found in the protocol article (39).

#### Clinical Diagnosis

Absence and history of MDD at the start of, and relapse and recurrence of MDD throughout study participation was assessed with the SCID-I for DSM-IV disorders (58) up to 12 weeks post-partum. Comorbidities on the Axis-I scale were assessed with the SCID-I as well.

#### Experience Sampling Methodology

The questions consisted of 25 items, including questions regarding positive and negative affect. The questions were derived from previous research (49) and are based upon the positive and negative affect schedule (PANAS) (61).^1^

#### Assessments

Participants' characteristics were assessed at baseline, including age, parity, and ADM usage. At baseline, and 24- and 36-weeks of pregnancy, the Edinburgh post-natal depression scale [EPDS (62)] for depressive symptoms, and the state-trait anxiety inventory [STAI (63)] for anxiety symptoms were assessed. The positive and negative affect scale [PANAS (61)] was used to assess different affective states of the participants at baseline, and 24- and 36-weeks of pregnancy. In addition, birth weight, gestational age and pregnancy-related complications were collected. In the RCT, birth outcomes were obtained from the reports from midwives and gynecologists. Participants in the cohort provided this information themselves during the 4 weeks post-partum assessment.

### Statistical Plan

To exploratively analyze differences in affect fluctuations during the first 8 weeks of study participation between the PCT while tapering ADM group and the continuation of ADM group, the ESM-trial data was used. An average positive affect and negative affect score was calculated for each reply to the trigger, minimizing the number of separate variables. That way, there was one score for each trigger for both positive and negative affect. Participants were excluded if they responded to <4 triggers in total, but were allowed to have missing data. Missing data was not imputed. To estimate individual affect fluctuations, an individual linear regression equation was calculated using participants' average scores on the positive and negative affect items. For this individual linear regression equation, the dependent variable was the mean affect score for each trigger, and elapsed time was the independent variable. The individual beta coefficient, the coefficient of determination (*R*^2^), and one minus *R*^2^ (as a measure of fluctuations) for both positive and negative affect were saved for each participant and used in subsequent analyses. The responses and individual regression lines were plotted for each individual, to visually inspect patterns of positive and negative affect fluctuations.

For the analyses, the group differences in positive and negative affect fluctuations (ESM-trial data), with and without correction for depressive symptoms (assessed with the EPDS), and number of recurrences were analyzed if feasible. Second, three separate linear regression analyses were conducted; to (1) predict depressive symptoms with ESM positive and negative affect fluctuations; (2) predict recurrences with ESM positive and negative affect fluctuations; and (3) predict birth weight with ESM positive and negative affect fluctuations corrected for pre-natal depressive symptoms. All regression analyses were corrected for number of previous episodes, as this was used to stratify the randomization in the RCT sample and is a known predictor of depressive recurrences (64).

## Results

Overall, 478 pregnant women were referred to the studies for counseling, of whom 146 were eligible to participate (*n* = 44 participated for the RCT; *n* = 102 for the observational cohort). Among these participants, 24 women agreed to participate in the ESM-trial. See Figure 1 for a flow-chart. Sufficient data from 19 women were available for the analyses, including 12 RCT participants of whom eight received PCT and tapered the ADM, and four continued ADM usage. Seven participants where from the observational cohort, of whom six continued the use of ADM, and one tapered without additional relapse prevention intervention. An overview of baseline characteristics is displayed in Table 1, and the study flow-chart including reasons for drop-out is shown in Figure 1. The results of the follow-up measurements are reported in Table 2.

**Table 1:** Baseline participant characteristics.

	**ESM-trial (*n* = 19)**	**RCT Stop (*n* = 24)**	**RCT Go (*n* = 20)**	**Cohort (*n* = 94)**
Mean age in years (*SD*)	32.3 (4.8)	31.6 (5.3)	31.4 (4.3)	31.3 (4.3)
Nulliparous (%)	2 (15)	3 (20)	1 (8)	7 (16)
Born in the Netherlands (%)^a^	18 (100)	22 (100)	14 (87)	89 (99)
Marital status				
Single (%)	1 (5.3)	1 (4)	1 (5)	3 (3)
Partner, living apart (%)	1 (5.3)	1 (4)	1 (5)	5 (5)
Married or cohabiting(%)	17 (89)	22 (92)	17 (89)	86 (91)
Smoking (%)	2 (10)	3 (12)	1 (5)	5 (5)
Education				
Primary school or Secondary education (%)	0 (0)	1 (4)	1 (5)	4 (4)
Vocational or Pre-university education (%)	8 (44)	11 (46)	8 (40)	27 (30)
Higher education (%)	10 (56)	12 (50)	11 (55)	58 (65)
Duration ADM usage, in months (*SD*)	72.9 (59.0)	56.0 (50.0)	50.6 (38.0)	72.3 (65.0)
Number of previous MDD episodes (*SD*)	1.9 (1.0)	2.2 (1.2)	1.9 (1.5)	1.9 (1.5)
Comorbid DSM-IV Axis-I disorders, yes (%)	7 (37)	9 (37)	7 (35)	28 (29)
Mean EPDS score (*SD*)	5.5 (3.3)	6.3 (3.6)	4.5 (3.1)	6.5 (4.4)
Mean STAI score				
STAI—state (*SD*)	33.3 (9.1)	34.0 (8.2)	32.6 (7.6)	35.2 (9.5)
STAI—trait (*SD*)	39.5 (7.8)	39.3 (7.1)	35.4 (6.5)	40.4 (9.6)
Mean PANAS score				
Positive affect (*SD*)	2.1 (0.6)	2.0 (0.6)	2.2 (0.7)	2.0 (0.7)
Negative affect (*SD*)	0.7 (0.7)	0.8 (0.7)	0.5 (0.5)	0.9 (0.7)

*Not all information is available for each participant, resulting in small variations of percentages. Numbers are rounded. RCT, Randomized controlled trial; RCT Stop, RCT group receiving preventive cognitive therapy while tapering ADM; RCT Go, RCT group continuing ADM; ADM, antidepressant medication; ESM, experience sampling methodology; HRSD-17, Hamilton Rating Scale for Depression−17 items; MDD, major depressive disorder; DSM-IV, Diagnostic and Statistical Manual of Mental Disorders fourth edition; EPDS, Edinburgh post-natal depression scale, STAI, state-trait anxiety inventory; PANAS, positive and negative affect scale*.

a *Chi-square indicates significant differences between RCT Stop, RCT Go, and Cohort group (p = 0.02)*.

**Table 2:** Group results follow-up measurements and EMA–trial.

**ESM-trial (*n* = 19)**	**RCT Stop (*n* = 8)**	**RCT Go (*n* = 4)**	**Cohort (*n* = 7)**
No. of recurrences	1	0	1
Mean number of responses (*SD*)	40.9 (34.4)	19.2 (13.9)	48.9 (27.4)
Positive affect			
*B*	0.07 (0.31)	0.25 (0.44)	−0.04 (0.12)
*R^2^*	0.09 (0.13)	0.19 (0.12)	0.03 (0.05)
Negative affect			
*B*	1.06 (3.15)	−0.25 (0.49)	0.10 (0.20)
*R^2^*	0.21 (0.26)	0.11 (0.18)	0.09 (0.13)
Stress	31.79 (14.16)	23.35 (11.65)	25.52 (31.82)
**Main trial results (*****n*** **=** **121)**	**RCT Stop (*****n*** **=** **19)**	**RCT Go (*****n*** **=** **19)**	**Cohort (*****n*** **=** **83)**
Mean birthweight, in gram (*SD*)	3,632.1 (474.3)	3,369 (422.9)	3,382.6 (463.2)
Mean gestational age, in days (*SD*)	277 (6.9)	275 (5.5)	275 (9.3)
Mean BW for GA, percentile (*SD*)	43.4 (33.7)	41.6 (31.2)	48.2 (29.8)

*Numbers are rounded. RCT, Randomized controlled trial; RCT Stop, RCT group receiving preventive cognitive therapy while tapering ADM; RCT Go, RCT group continuing ADM; ADM, antidepressant medication; ESM, experience sampling methodology; BW, birthweight; GA, gestational age; Mean BW for GA, birthweight corrected for gestational age*.

### ESM-Trial Results (*n* = 19)

**Figure 2:**
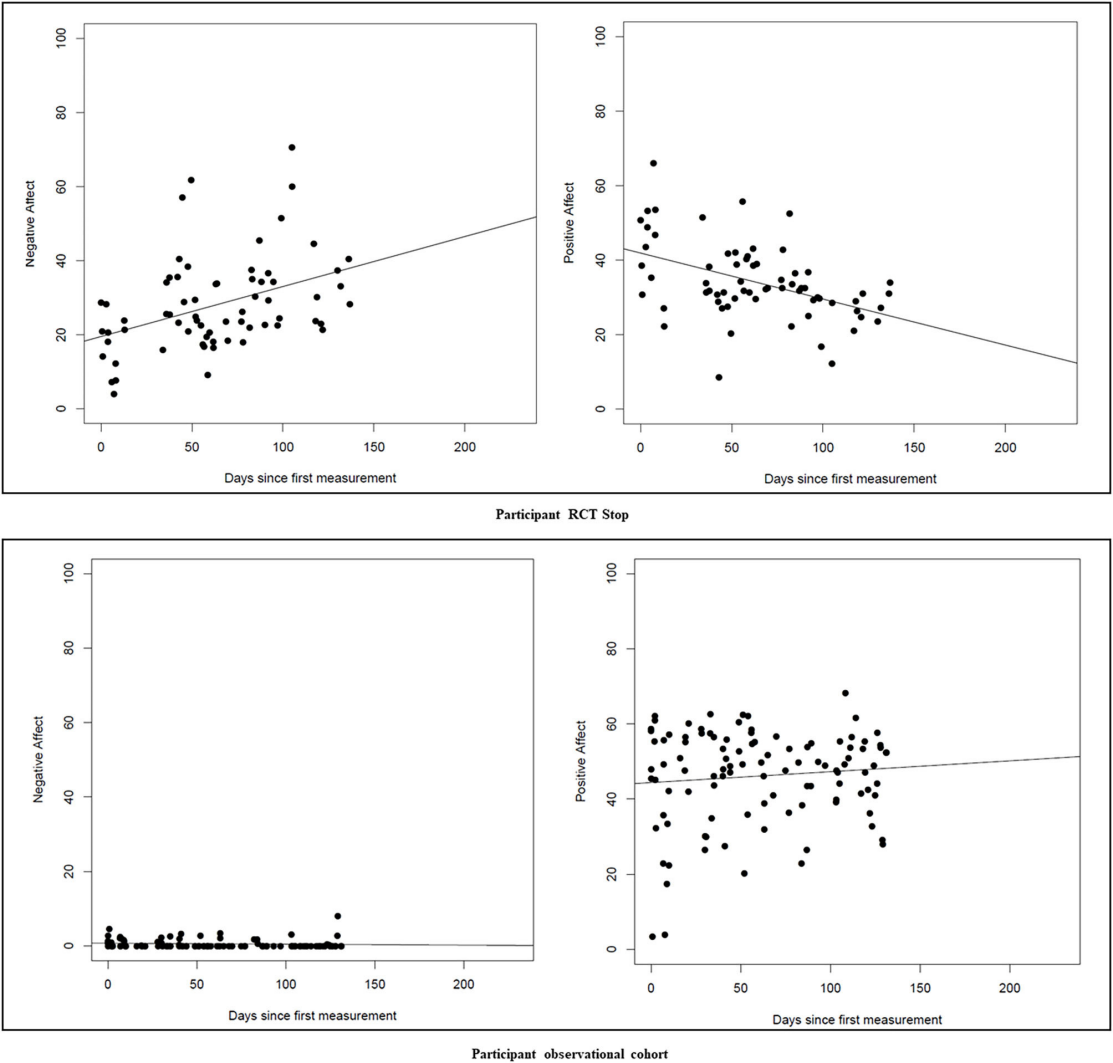
Two participant examples of positive and negative affect fluctuations in the ESM-trial.

In total, 2 out of 19 women participating in the ESM-trial had a recurrence of MDD during the study period. Mean positive affect score across groups was 48.56 (*SD* = 16.61), and mean negative affect was 20.51 (*SD* = 13.35). There was no significant group effect (RCT women tapering ADM vs. RCT women continuing ADM) on positive or negative affect fluctuations (positive affect: *F*_(1, 9)_ = 1.85, *p* = 0.20; negative affect: *F*_(1, 9)_ = 0.44, *p* = 0.52). When including women from the observational cohort into the ESM-trial analyses, this effect between tapering and continuing ADM remained non-significant (positive affect: *F*_(1, 15)_ = 0.16, *p* = 0.69; negative affect: *F*_(1, 15)_ = 3.86, *p* = 0.07). Correcting the analyses for depressive symptoms at baseline did not change the main results (positive affect: *F*_(1, 14)_ = 0.21, *p* = 0.65; negative affect: *F*_(1, 14)_ = 3.45, *p* = 0.08). As an example of the ESM-trial outcomes, Figure 2 displays the positive and negative affect scores for a pregnant woman who received PCT and tapered ADM (RCT participant), and a pregnant woman who continued ADM (cohort participant).

Linear regression analyses indicated that positive or negative affect fluctuations did not significantly predict depressive symptoms at 36 weeks pregnancy (positive affect β = 0.22, 95% *CI* = −15.84, 37.20, *p* = 0.40; negative affect β = −0.26, 95% *CI* = −22.55, 7.96, *p* = 0.32). Furthermore, there was no significant relationship between affect fluctuations and birth weight (positive affect β = −0.08, 95% *CI* = −3,083.36, 2,310.35, *p* = 0.76; negative affect β = 0.18, 95% *CI* = −1,081.73, 2,052.82, *p* = 0.51) or corrected birth weight for gestational age (positive affect β = 0.17, 95% *CI* = −120.28, 217.74, *p* = 0.54; negative affect β = 0.18, 95% *CI* = −67.96, 128.48, *p* = 0.52). There was an insufficient number of recurrences in the ESM-trial to investigate and test the relationship between affect fluctuations and recurrences of MDD.

### Main RCT and Cohort Results

In the main trials (RCT and observational cohort), 13 women had a recurrence of MDD during the study period (8.9%). The number of recurrences did not significantly differ between the groups in the main RCT (PCT with tapering ADM *n* = 3 vs. ADM continuation *n* = 3), but did in the full group (taper ADM with or without PCT *n* = 8 vs. ADM continuation *n* = 5; *X*^2^(1) = 8.60, *p* = 0.003).

## Discussion

The aim of the current study was to explore the effect of tapering ADM in pregnant women while receiving PCT as compared to pregnant women continuing ADM. In this proof-of-principle study, there were no indications that pregnant women who received PCT and tapered ADM showed more affect fluctuations or recurrences than women continuing ADM. To our knowledge this is the first study to show individual fluctuations of positive and negative affect and depression recurrence rates in an RCT and observational cohort of pregnant women using ADM at the start of their pregnancy.

Although the study had an explorative nature with a relatively small group of women, there was a large amount of individual responses (ranging from 6 to 105 responses per individual). The results of the ESM-trial indicate that women receiving PCT while tapering ADM show similar patterns of fluctuations in positive and negative affect throughout pregnancy, as women continuing ADM. This may be due to the effects of PCT which targets cognitive vulnerabilities by challenging dysfunctional beliefs and schema's, activating positive networks, and enhancing the memory of positive experiences (28). PCT therefore partially targets emotion regulation (28), by changing the way a person processes and regulates emotion-related information (65). In addition, a previous study showed that people who received PCT were less affected by daily hassles, which may have led to a reduced risk of recurrence in comparison to care as usual (66). Potentially, women receiving this preventive psychotherapy therefore did not show increased fluctuations in their affect or inertia, as they were able to regulate their emotions and/or process daily hassles better, thereby potentially lowering the risk of recurrence of MDD.

This is in contrast to a previous study among formerly depressed remitted participants that showed that people receiving PCT while tapering ADM had a slightly higher risk of recurrence in the first 4 months of tapering, indicating there may have been a withdrawal effect in these participants due to various reasons [e.g., imbalance due to withdrawal symptoms, or fear of recurrence; (27)]. In the current study, there was no difference in the number of recurrences, as well as no difference in positive and negative affect fluctuations in the group of women receiving PCT while tapering ADM. This might imply that there was none or less emotional withdrawal or discontinuation symptoms (67, 68), or fear of recurrences, in this group. An explanation for this difference may be that women learned to regulate emotions with the help of PCT (28, 65). The ESM-trial was conducted during the time that women tapered ADM and received PCT. Alternatively, unknown factors caused by the pregnancy itself might have protected the women from fluctuating more in affect while tapering ADM vs. continuation of ADM. This alternative explanation is supported by the fact that the few recurrences of depression in the main RCT primarily occurred in the first 3 months post-partum.

Another potential explanation for the absence of significant differences in affect fluctuations between PCT while tapering ADM vs. continuation of ADM, may be the low recurrence rates among the participants, with no significant differences in recurrence rates between women receiving PCT while tapering ADM and women continuing ADM. Previous studies investigating recurrence rates in pregnant women discontinuing ADM are scarce and have produced conflicting results. Although previous research found even lower recurrence rates in a group of pregnant women with a history of MDD [2.5% (69)], another study showed an increased risk of recurrence after pre-natally tapering ADM compared to continuation of ADM [68% (3)]. Yet another study found comparable recurrence rates in both groups [16% (6)]. One of the main differences between these studies is the selection of specific populations using different prevention strategies. For example, the pregnant women from previous studies had a history of more severe MDD and/or more or severe comorbid psychopathology, tapered ADM without guidance or complementary psychotherapy, or were actively seeking help and therefore may have had subsyndromal depressive symptoms for which they needed help. In contrast, the current study focused on the results of the RCT and cohort with stable remitted previously depressed pregnant women.

There were also no differences in the birth weight of offspring from women tapering or continuing ADM. Birth weight may already be affected by ADM use in early pregnancy, as previous reviews indicated (23). On the other hand, psychotherapy may likewise positively or negatively influence birth weight. A recent meta-analysis found that birth weight could be negatively or positively affected when pregnant women with common mental disorders received psychotherapy, depending on the disorder and type of treatment (16). The absence of a direct influence of affect fluctuations on birth weight does not necessarily mean that these fluctuations do not affect the offspring. It may be the case that adverse effects are expressed later in life. For example, one study showed that levels of maternal mood symptoms during pregnancy predicted negative affect in the offspring at age one, two, and seven (55), and another study demonstrated that elevated anxiety symptoms increased the risk of offspring having delayed development at the age of 3 years (54). Alternatively, the absence of a link between affect fluctuations and birth weight can be explained by the small sample size.

Despite several strengths of the current study, being the first proof-of-principle ESM RCT to investigate tapering ADM in pregnant women while receiving a psychotherapeutic intervention as compared to continuation of ADM, there were several limitations that need to be addressed. First, the small sample size in the ESM-trial prevented us from drawing firm conclusions about the potential role of affect fluctuations. However, 800 datapoints have been gathered enabling us to analyse continuous fluctuations in affect with a minimal risk of recall bias [e.g., (57)], and more details regarding the effect of tapering ADM with PCT among pregnant women. Second, the low recurrence rates and low variation of depressive symptoms throughout pregnancy may have minimized the likelihood of detecting clinically meaningful associations. However, low recurrence rates while tapering ADM might also indicate that tapering ADM in pregnancy has limited risks while receiving PCT. Lastly, the participant group may have comprised relatively healthy women with low levels or residual depressive symptoms, which may have put them at low risk of recurrence. Although, our sample at baseline on average had two previous MDD episodes, comparable to other depression-related study samples. Due to these limitations, the results of this proof-of-principle study must be interpreted with caution, and a replication of the study is needed.

Overall, the current study and explorative analyses provide no first indications that pregnant women show more fluctuations in positive/negative affect, nor recurrences, when they taper ADM and receive PCT compared to when women continue ADM. This is supported by the results of the main trial, where the pregnant women in both groups had a comparable risk of relapse (41). Pregnant women may not be at increased risk of recurrence when they taper ADM, or put their child at risk of negative outcomes such as lower birth weight. Future research should explore recurrence risk in larger (international) samples and individual pathways of affect fluctuations and MDD recurrence in pregnant women with a history of MDD who use ADM. Moreover, the effects and effectiveness of relapse prevention treatments for both mother and child need to be further investigated. More research is needed to investigate whether indeed the PCT led to the current results.

To conclude, the current proof of principle study provided a first indication that tapering ADM with the guidance of PCT may indeed protect pregnant women against recurrence of depression and affect fluctuations, without evident negative effects on birth weight of the offspring.

## Data Availability Statement

The raw data supporting the conclusions of this article will be made available by the authors, without undue reservation.

## Ethics Statement

The studies involving human participants were reviewed and approved by Medical Ethical Committee of the Erasmus Medical Center Rotterdam, the Netherlands. The patients/participants provided their written informed consent to participate in this study.

## Author Contributions

MB, NM, CB, ML, and HB: design, protocol, and data acquisition. CA, MB, CB, and HB: analyses. MB, NM, CB, HB, AW, ML, and CA: interpretation of data, drafting of the manuscript, and/or revisions. All authors contributed to the article and approved the submitted version.

## Conflict of Interest

CB was co-developer of the Dutch multidisciplinary clinical guideline for anxiety and depression, for which she received no remuneration. CB was furthermore a member of the scientific advisory board of the National Insurance Institute, for which she received an honorarium. This role had no direct relation to this study. CB received royalties from her books and co-edited books, and she developed Preventive Cognitive Therapy on the basis of the Cognitive Model of A. T. Beck. The remaining authors declare that the research was conducted in the absence of any commercial or financial relationships that could be construed as a potential conflict of interest.

## Footnotes

^1^A full overview of the ESM micro-study questions is available upon request.
